# Association between the Dental Check-Ups Prevalence and Percentage of Inhabitants with All Natural Teeth

**Published:** 2019-01

**Authors:** Marian MARAK, Pavol BENO, Martin SAMOHYL

**Affiliations:** 1. Faculty of Social Sciences, University of Ss. Cyril and Methodius, Trnava, Slovakia; 2. Department of Laboratory Medicine, Faculty of Health Sciences and Social Work, Trnava University, Trnava, Slovakia; 3. Institute of Hygiene, Faculty of Medicine, Comenius University, Bratislava, Slovakia

## Dear Editor-in-Chief

Smiling, chewing, speaking, biting and psychosocial wellbeing have impact on oral quality of life (1). Loss teeth causes can be e.g. dental injuries, periodontal disease (2), poor oral hygiene (caries), opiate drug (3), dietary changes (4) and smoking. The aim of the study was to analyze association between the dental check-ups prevalence and percentage of inhabitants with all natural teeth in 27 European Union member countries.

The data were analysed from database of Euro-barometer 72.3 (5). The Eurobarometer questionnaires (respected national language) was collected from 30.292 citizens (27 European Union member countries; n=26993 inhabitants).

**Fig. 1:**
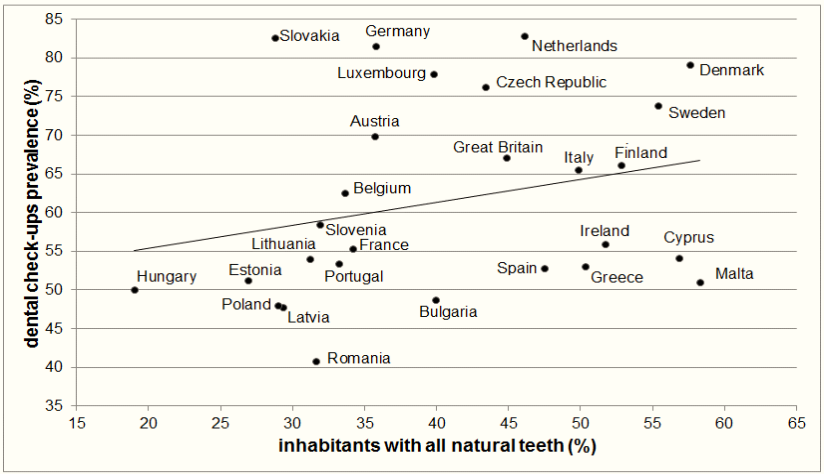
Association between the dental check-ups prevalence (%) and percentage of inhabitants with all natural teeth by 27 European Union member countries, the latest available data (source: own processing of data from Eurobarometer 72.3, Public Health Attitudes, Behavior, and Prevention, 2009)

Dental check-ups prevention was taken on own initiative in 8.3% inhabitants, on doctor's initiative in 25.0% inhabitants, in screening programme in 8.6% inhabitants and in 58.0% inhabitants’ did not take a dental check-ups prevention in the Slovak Republic. The most inhabitants had ≥ 20 natural teeth (42.0%) and all natural teeth had 28.8% inhabitants in the Slovak Republic. The highest dental check-ups prevalence was in Netherlands (82.8%) and the most inhabitants with all natural teeth were in Malta (58.3%). Between the dental check-ups prevalence and percentage of inhabitants with all natural teeth by country was found positive correlation (Pearson's r = 0.36) (Fig. 1).

In the second place from 27 European Union member countries in dental check-ups prevalence was the Slovak Republic (82.6%), where dental doctors, nursery, schools, the Regional Public Health Authorities give emphasis on dental education and dental programs for children pre-school age and school age. The Slovak Republic has the third the lowest percentage of inhabitants with all natural teeth (28.8%) from 27 European Union member countries. It can be partially explained by high prevalence of sweet foods consumption and sweet drinks consumption. Sweet foods and sweet drinks are often consumed (20.1% of inhabitants) in the Slovak Republic (5).

## References

[1] Tsai S-J Lin M-S Chiu W-N (2015). Factors associated with having less than 20 natural teeth in rural adults: a cross-sectional study. BMC Oral Health, 15: 158. 26654530 10.1186/s12903-015-0147-y PMC4676875

[2] Cavalcanti AL Ramosis IA Cardoso AMR (2016). Association between Periodontal Condition and Nutritional Status of Brazilian Adolescents: A Population-based Study. Iran J Public Health, 45 ( 12): 1586– 1594. 28053924 PMC5207099

[3] Shekarchizadeh H Khami MR Mohebbi SZ (2013). Oral Health of Drug Abusers: A Review of Health Effects and Care. Iran J Public Health, 42 ( 9): 929– 940. 26060654 PMC4453891

[4] Ariga P Bridgitte A Rangarajan V (2012). Edentulousness, Denture Wear and Denture Needs of the Elderly in Rural South India. Iran J Public Health, 41( 7): 40– 43. PMC3469015 23113208

[5] European Commission (2009). Eurobarometer 72.3: Public Health Attitudes, Behavior, and Prevention . Inter-university consortium for political and social research (ICPSR). http://www.icpsr.umich.edu/icpsrweb/ICPSR/studies/32441

